# Benchmarking network-based gene prioritization methods for cerebral small vessel disease

**DOI:** 10.1093/bib/bbab006

**Published:** 2021-02-26

**Authors:** Huayu Zhang, Amy Ferguson, Grant Robertson, Muchen Jiang, Teng Zhang, Cathie Sudlow, Keith Smith, Kristiina Rannikmae, Honghan Wu

**Affiliations:** Centre for Medical Informatics, Usher Institute, University of Edinburgh, Edinburgh, United Kingdom; Centre for Medical Informatics, Usher Institute, University of Edinburgh, Edinburgh, United Kingdom; Institute for Adaptive and Neural Computation, School of Informatics, University of Edinburgh, Edinburgh, United Kingdom; Edinburgh Medical School, University of Edinburgh, Edinburgh, United Kingdom; Department of Orthopaedics and Traumatology, the University of Hong Kong, Hong Kong, China; Centre for Medical Informatics, Usher Institute, University of Edinburgh, Edinburgh, United Kingdom; Health Data Research UK, London, United Kingdom; Centre for Medical Informatics, Usher Institute, University of Edinburgh, Edinburgh, United Kingdom; Health Data Research UK, London, United Kingdom; Centre for Medical Informatics, Usher Institute, University of Edinburgh, Edinburgh, United Kingdom; Health Data Research UK, London, United Kingdom; Health Data Research UK, London, United Kingdom; Institute of Health Informatics, University College London, London, United Kingdom

**Keywords:** network-based gene prioritization, cerebral small vessel disease, protein–protein interaction, disease gene association, benchmarking

## Abstract

Network-based gene prioritization algorithms are designed to prioritize disease-associated genes based on known ones using biological networks of protein interactions, gene–disease associations (GDAs) and other relationships between biological entities. Various algorithms have been developed based on different mechanisms, but it is not obvious which algorithm is optimal for a specific disease. To address this issue, we benchmarked multiple algorithms for their application in cerebral small vessel disease (cSVD). We curated protein–gene interactions (PGIs) and GDAs from databases and assembled PGI networks and disease–gene heterogeneous networks. A screening of algorithms resulted in seven representative algorithms to be benchmarked. Performance of algorithms was assessed using both leave-one-out cross-validation (LOOCV) and external validation with MEGASTROKE genome-wide association study (GWAS). We found that random walk with restart on the heterogeneous network (RWRH) showed best LOOCV performance, with median LOOCV rediscovery rank of 185.5 (out of 19 463 genes). The GenePanda algorithm had most GWAS-confirmable genes in top 200 predictions, while RWRH had best ranks for small vessel stroke-associated genes confirmed in GWAS. In conclusion, RWRH has overall better performance for application in cSVD despite its susceptibility to bias caused by degree centrality. Choice of algorithms should be determined before applying to specific disease. Current pure network-based gene prioritization algorithms are unlikely to find novel disease-associated genes that are not associated with known ones. The tools for implementing and benchmarking algorithms have been made available and can be generalized for other diseases.

## Introduction

‘Guilt by association’ is the most adopted concept in network-based gene prioritization methods. The underlying principle is that genes that are closely associated in the protein–gene interaction (PGI) network tend to be in the same functional module, thereby giving rise to similar phenotypes [1]. Different algorithms have been developed and applied to biological interaction networks under this principle. These algorithms take a set of known genes associated with a disease (seed genes) as input and try to predict or prioritize other potential genes associated with the disease. Network propagation algorithms were among the 1st algorithms to be applied on the PGI network in the form of a random walk with restart (RWR) algorithm [2]. Despite its early application and simplicity in a theoretical and computational sense, it showed superior or as good performance to many algorithms and was often taken as a reference algorithm [3–5]. The RWR algorithm was later extended to work on the disease–gene heterogeneous network by either directly expanding the adjacency matrix (RWRH) [6] or allowing propagation on both the protein/gene network and disease similarity network (IDLP) [4]. Some other algorithms, like DIAMoND and GenePanda, find special associations between candidate genes and seed genes using defined heuristic rules[7, 8]. Recently, the network embedding method Node2Vec (N2V) has also been used in gene prioritization [9, 10].

However, the reports describing the algorithms typically showcased their performance in an example disease or condition, so that it is not clear for end users who wish to apply the algorithms to the disease of their interest which algorithm is the optimal one. To address this issue, we benchmarked seven representative algorithms for their application in non-amyloid cerebral small vessel disease (hereafter referred to as cSVD). CSVD is a term used to describe a variety of pathological processes that affect the deep small penetrating arteries, arterioles, venules and capillaries of the brain. The main clinical phenotypes of cSVD include small vessel ischemic stroke, deep intracerebral haemorrhage and vascular cognitive impairment [11, 12]. The overall burden of cSVD is growing as the world’s population continues to age [13]. Other than management of hypertension, we currently lack effective treatments to reduce the risk of cSVD. Hence, pathways involved in cSVD pathogenesis must be better understood to develop new effective prevention and treatment strategies. Genetic studies may offer an opportunity for further insights.

In this article, we performed domain knowledge-lead curation of PGIs and disease–gene associations to assemble the input network. Known cSVD-associated genes summarized from a systematic review of familial cSVD were taken as seed genes [14]. We accessed the performance of representative network-based gene prioritization algorithms with cross-validation. The candidate genes prioritized by best performing algorithms were externally evaluated with results of genome-wide association study (GWAS) MEGASTROKE [15, 16].

## Methods

**Figure 1:**
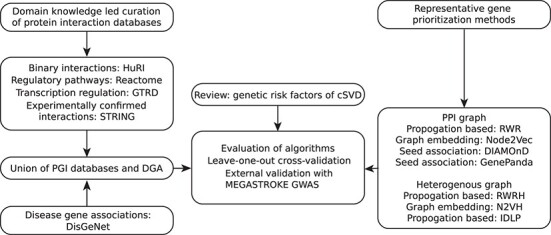
Benchmarking workflow of network-based gene prioritization in cSVD. There are three main components of the benchmarking workflow: assembling input networks, selection of algorithms and validation of algorithms. (i) PGI was assembled with domain knowledge-lead curation of protein/gene interactions from four databases. DGAs were added to PGI to generate disease–gene heterogeneous networks. (ii) Representative gene prioritization algorithms were selected based on the originality with the non-network-based algorithms or hybrid algorithms excluded. (iii) Performance of algorithms was assessed with LOOCV and externally validated with MEGASTROKE GWAS results. Abbreviations: PGI, protein–gene interaction; DGA, disease–gene association; cSVD, cerebral small vessel disease; LOOCV, leave-one-out cross-validation; GWAS, genome-wide association study. Please see Table 1 for full names of databases and Table 2 for full names of algorithms.

The benchmarking pipeline is in three main parts: curation of PGI and disease–gene networks, implementation of algorithms and evaluation of algorithm performances (Figure 1).

### Sources of data used as input to the network

For curation of human PGIs, three overall preferences on the nature of databases were pursued with descending priority: (i) coverage of seed genes (reviewed by Rannikmäe *et al.* [14]), (ii) the objectivity of database and (iii) presence of experimental evidence to support the interaction. In addition, we made sure that seed genes were covered in at least one of the databases, so that algorithms could use this prior information to prioritize other candidate genes.

Objectivity signified to what extent relationships found for each protein or gene were not affected by the researchers’ interests. Databases curating binary protein interactions determined by yeast-2-hybrid screening are good examples of data sources with high objectivity, since neither proteins of interest nor relationships to be observed are preselected. Databases curating transcription regulation defined by chromatin immunoprecipitation sequencing (ChIP-Seq) are examples of moderate objectivity, since specific transcription factors are chosen to be studied, but the regulated genes were accessed universally with RNA sequencing. Both the objectivity and experimental evidence requirements implied the exclusion of relationships extracted by literature text-mining methods. An overview of all databases curated is provided in Table 1.

**Table 1:** Summary information on PGIs and disease–gene associations curated from different data sources

**Database**	**Gene/protein**	**Disease**	**Node**	**Interaction**	**Selection/filter**
HuRI^a^	8327	–	8327	19 082	HuRI
GTRD^b^	8275	–	8275	52 569	Promotor (−1000, +1000); more than 8 binding sites per gene
Reactome	5219	–	5219	29 328	–
String	13 444	–	13 444	91 019	Experimentally confirmed with score ≥350
DisGeNET	7635	8431	16 066	67 993	DGA score ≥0.3
Mimminer	–	2646	2646	9840	Similarity score >0.6
Gene network	18 718	–	18 718	183 457	–
Disease–gene network	19 463	10 103	29 566	261 298	–

^a^HuRI—Human Reference Interatome

^b^GTRD—Gene Transcription Regulation Database

Binary interactions (protein interaction determined by yeast two-hybrid screening) were curated from the Human Reference Interactome (HuRI) database [17]. Transcription regulations were curated from the Gene Transcription Regulation Database (GTRD) [18]. Regulations with more than eight (including eight) binding sites determined by peak calling of ChIP-Seq signal in the genomic range of 1000 bp up- or down-stream of transcription start site were selected. Relationships in biological pathways were curated from Reactome databases [19]. To cover all the seed genes, additional experimental confirmed relationships were curated from the String database with a filter of confidence score ≥350 (Table 1) [20]. Since curation of GTRD database required both gene and protein entities on the graph, we created a hybrid protein–gene network. All entities in PGI were converted to Ensembl gene ID to allow best compatibility with gene–disease associations (GDAs).

GDAs were curated from DisGeNet v6.0 using the ‘ALL gene-disease association’ file [21]. Selected associations were confined to human evidence with associations GDA scores ≥0.3, which corresponded to associations curated from evidence-based databases. The disease similarity (Dsim) score was extracted from Mimminer [22]. Diseases with similarity score greater than 0.6 were given an edge in the network. All disease entities were mapped to ids of the Online Mendelian Inheritance in Man database.

Unions of all PGIs with or without GDAs were computed and non-directed simple networks were generated (Table 1). The network edge lists and code to extract the relationships were published at https://github.com/huayu-zhang/gp-bench.

### Modularity of GO pathways

Clustering of genes in the same pathways is a known property of PGI networks. To test whether our curated PGI network had this property, we extracted groups of genes defined by gene ontology (GO) terms of biological process and calculated modularity of GO pathways on the PGI network. Modularity quantifies if the number of edges among a group of nodes (modules) is lower or higher than expected. The modularity of GO pathways was calculated as a two-community modularity with one community defined by a GO pathway and the other community being the rest of nodes:

(1.1) \begin{equation*} Q=\frac{1}{4m}{\boldsymbol{s}}^{\boldsymbol{T}}\boldsymbol{Bs} \end{equation*}

(1.2) \begin{align*} {B}_{ij}=\left\{\begin{array}{cc}{A}_{\boldsymbol{ij}}-\frac{k_i{k}_j}{2m}& if\ i\ne j\\{}0& if\ i=j\end{array}\right. \end{align*}

(1.3) \begin{equation*} {s}_i=\left\{\begin{array}{cc}-1& if\ i\in GO\ pathway\\{}1& if\ i\notin GO\ pathway\end{array}\right. \end{equation*}

where $Q$ is the modularity score, $m$ is the number of edges and ${k}_i$ and ${k}_j$ are the degrees of $i$-th and $j$-th nodes.

### Graph-based gene-prioritization methods

#### Algorithm selection

To select algorithms for comparison, a PubMed search for ‘network-based gene prioritization’ was done and 49 articles were yielded. Additional 77 articles were obtained through the review of Zolotareva and Kleine [23], 51 of which were excluded since it was reviewed to be not available. The 75 articles were screened. Non-network-based algorithms or hybrid algorithms combining network-based approaches and machine learning approaches were excluded to focus on the network-based algorithms and improve comparability among algorithms. We also excluded articles if only implementations but not the original algorithms were described or if the source code was not provided for redevelopment. The selection resulted in 35 articles describing different algorithms, among which algorithms with similar core mechanisms exist. To avoid redundantly testing similar algorithms, seven representative algorithms with different mechanisms were selected for benchmarking. Details of algorithm selection process are given in Supplementary Table S1. A summary of the selected algorithms is given in Table 2.

**Table 2:** Summary of network-based gene prioritization methods applied in this study

**Abbreviation**	**Name**	**Mechanism**	**Network**
RWR	Random walk with restart	Network propagation	Gene–protein network
N2V	Node2Vec	Graph embedding	Gene–protein network
DIAMOnD	Disease module detection	Seed association	Gene–protein network
GenePanda	GenePanda	Seed association	Gene–protein network
RWRH	RWR on heterogeneous network	Network propagation	Disease–gene network
N2VH	N2V on heterogeneous network	Graph embedding	Disease–gene network
IDLP	Improved dual label propagation	Network propagation	Disease–gene network

#### Notations

For describing the methods, common notations were used. The PGI network $\boldsymbol{G}=(\boldsymbol{V},\boldsymbol{E})$ consists of a node set, $\boldsymbol{V}$, of $n$ nodes, representing individual proteins/genes, and an edge set, $\boldsymbol{E}$, of node pairs, representing PGIs. The set of seed nodes was represented by $\boldsymbol{S}$, which contained monogenic risk factors of cSVD. The number of elements in a set is notated with single vertical bars (e.g. $\Big|\boldsymbol{S}\Big|$ for number of seed nodes). The network of $\boldsymbol{G}$ is represented by an $n\times n$ adjacency matrix $\boldsymbol{A}$, and the column-normalized adjacency matrix was represented by $\boldsymbol{W}$.

(2.1) \begin{equation*} \boldsymbol{W}=\boldsymbol{A}{\boldsymbol{D}}_{\boldsymbol{k}}^{-\mathbf{1}} \end{equation*}

where ${\boldsymbol{D}}_{\boldsymbol{k}}$ is a diagonal matrix ${\boldsymbol{D}}_{\boldsymbol{k}}=\mathit{\operatorname{diag}}(\boldsymbol{k})$ and $\boldsymbol{k}$ is a vector in which the $i$-th elements is the degree of $i$-th node. Adjacency matrix for GDA network and disease similarity network were notated with ${\boldsymbol{A}}_{\boldsymbol{PD}}$ and ${\boldsymbol{A}}_{\boldsymbol{DD}}$. Bold font was used for notations of vectors and matrices.

Since the output of different algorithms are not directly comparable, we also described in the following section how node ranks were generated for each algorithm. To streamline the comparisons of algorithms, we implement all algorithms in Python 3.7 (https://github.com/huayu-zhang/gp-bench). A summary of the algorithms is given in Table 2.

#### Random walk with restart

RWR algorithm was first applied to the human PGI networks by Kohler *et al.* [2]. It has since been extended to work on the disease–gene heterogeneous network [6]. Intuitively, random walk measures the probability of ending on a particular node if one starts from the seed nodes. The probability can in turn be interpreted as a measure of distance from seed nodes with the network structure taken in consideration. We briefly describe the principles here. The RWR algorithm is defined as follows:

(2.2) \begin{equation*} {\boldsymbol{p}}_{\boldsymbol{t}}=\left(1-r\right)\boldsymbol{W}{\boldsymbol{p}}_{\boldsymbol{t}-\mathbf{1}}+r{\boldsymbol{p}}_{\mathbf{0}} \end{equation*}

The initial probability is ${p}_{0i}=1/\Big|\boldsymbol{S}\Big|$, if $i$-th node is one of the seed nodes; otherwise, ${p}_{0i}=0$. The restart probability $r$ was tuned in range of (0.1–0.9) with steps of 0.2. The process was repeated until convergence with a practical tolerance of difference ${\Big\Vert{\boldsymbol{p}}_{\boldsymbol{t}}-{\boldsymbol{p}}_{\boldsymbol{t}-\mathbf{1}}\Big\Vert}_1<{10}^{-8}$. Elements in the converged ${\boldsymbol{p}}_{\boldsymbol{t}}$ were used as the score for ranking all genes:

(2.3) \begin{equation*} \boldsymbol{RWR}\ \boldsymbol{score}={\boldsymbol{p}}_{\infty } \end{equation*}

For random walk on heterogeneous network (RWRH), the adjacency matrix $\boldsymbol{A}$ and probability vector ${\boldsymbol{p}}_{\mathbf{0}}$ and ${\boldsymbol{p}}_{\boldsymbol{t}}$ were expanded to accommodate disease–gene association and disease similarities:

(2.4) \begin{equation*} {\boldsymbol{A}}^{\boldsymbol{expand}}=\left[\begin{array}{cc}\boldsymbol{A}& {\boldsymbol{A}}_{\boldsymbol{PD}}\\{}{{\boldsymbol{A}}_{\boldsymbol{PD}}}^{\boldsymbol{T}}& {\boldsymbol{A}}_{\boldsymbol{DD}}\end{array}\right] \end{equation*}

(2.5) \begin{equation*} {\boldsymbol{p}}_{\boldsymbol{t}}^{\boldsymbol{expand}}=\left[\begin{array}{c}{\boldsymbol{p}}_{\boldsymbol{t}}\\{}\ {\boldsymbol{p}}_{\boldsymbol{t}}^{\boldsymbol{disease}}\end{array}\right] \end{equation*}

where ${\boldsymbol{p}}_{\boldsymbol{t}}^{\boldsymbol{disease}}$ is the probability vector for all disease nodes. The expansion allows random walk on both gene/protein nodes and disease nodes.

Node2Vec

N2V is a network embedding algorithm invented by Grover and Leskovec [10], which computes a low-dimensional vector representation for all nodes in a network. Full theoretical background is not repeated here. Briefly, the vector representation for each node is optimized in the way that the conditional log-probability of observing a network neighbourhood (sampled by random walks described below) is maximized. In other word, nodes with similar vector representations are likely from similar neighbourhood in the network, allowing us to find genes closely related to seed genes. Practically, for each node in $V$, neighbourhood sampling was done by generating ${n}_{walks}$ random walks with length ${l}_{walks}$. The number of walks ${n}_{walks}$ was tuned in values of (20, 40, 80), while length of walks ${l}_{walks}$ was tuned in values of (40, 80, 160). The balance between breadth-first search (BFS) and depth-first search (DFS) was controlled by $p$ (smaller $p$ favours BFS) and $q$ (smaller $q$ favours DFS). Both $p$ and $q$ were tuned in values of (0.5, 1, 2). The walks were then used as the input for *Word2Vec*, where each walk was treated as a sentence and each node was treated as a word. Using Skip-gram architecture, vectorized representation ${v}_i$ was computed for each node $i$. The dimension of the vectors $d$ was tuned in values of (64, 128, 256). Max cosine similarity of a node to seed nodes was used as the gene-prioritization score of N2V and was used for ranking candidate genes:

(2.6) \begin{equation*} N2V\ {score}_i=\mathit{\max}\left\{\frac{{\boldsymbol{v}}_{\boldsymbol{i}}\bullet{\boldsymbol{v}}_{\boldsymbol{s}}}{{\left\Vert{\boldsymbol{v}}_{\boldsymbol{i}}\right\Vert}_2{\left\Vert{\boldsymbol{v}}_{\boldsymbol{s}}\right\Vert}_2},s\in \boldsymbol{S}\right\} \end{equation*}

The N2V algorithm could also be applied to heterogeneous network without modification (N2VH).

*Disease module detection algorithm*

Disease module detection algorithm (DIAMOnD) was proposed by Ghiassian *et al.* [7]. The core mechanism of the DIAMOnD algorithm is stepwise inclusion of neighbour nodes of seed nodes based on hypergeometric distribution probability. The probability quantifies likelihood of observing certain number of connections to seed nodes based on the degree of the node. A lower probability suggests overrepresentation of connections to seed nodes. At the end of each step, the set of seed nodes is updated by the newly prioritized candidate node. For each candidate node at $t$-th step, probability of any candidate node connecting exactly to certain number of seed nodes is calculated based on the hypergeometric distribution:

(2.7) \begin{equation*} p=\frac{\left(\begin{array}{c}\left|{S}_t\right|\\{}{k}_{st}\end{array}\right)\left(\begin{array}{c}n-\left|{S}_t\right|\\{}k-{k}_{st}\end{array}\right)}{\left(\begin{array}{c}n\\{}k\end{array}\right)} \end{equation*}

where $k$ is the degree of the candidate node, ${S}_t$is the set of seed nodes at $t$-th step and${k}_{st}$ is the number of connections of the candidate node to ${S}_t$. The candidate node with lowest $p$ is prioritized and is incorporated in the list of seed node. The rank of nodes was given by the order of being selected in this stepwise gene prioritization process.

An extension which add additional weight to the original seed genes was given:

(2.8) \begin{equation*} p=\frac{\left(\begin{array}{c}\left|{S}_t\right|+\left(\alpha -1\right)\left|S\right|\\{}{k}_{st}+\left(\alpha -1\right){k}_s\end{array}\right)\left(\begin{array}{c}n-\left|{S}_t\right|\\{}k-{k}_{st}\end{array}\right)}{\left(\begin{array}{c}n+\left(\alpha -1\right)\left|S\right|\\{}k+\left(\alpha -1\right){k}_s\end{array}\right)} \end{equation*}

where ${k}_s$ is the number of connections of the candidate node to $S$ and $\alpha$ ($\alpha >1$) is the hyperparameter controlling the weight. The hyperparameter $\alpha$ was tuned in values of (1, 10, 100).

#### GenePanda

GenePanda was proposed by Yin *et al.* [8]. Briefly, in the GenePanda algorithm, the degree-adjusted distance ${d}_{ij}^{adj}$ between $i$-th node to $j$-th node is calculated:

(2.9) \begin{equation*} {d}_{ij}^{adj}={d}_{ij}/\sqrt{k_i{k}_j} \end{equation*}

where ${d}_{ij}$ is the shortest path length between $i$-th node to $j$-th node and ${k}_i$ and ${k}_j$ are degrees of $i$-th node to $j$-th node. The GenePanda score is defined as the difference of average adjusted distance of a node to the whole network to the average adjusted distance to the seed genes. The GenePanda score for $i$-th node is calculated as follows:

(2.10) \begin{equation*} {GenePanda\ score}_i=\frac{\sum_{j\in \boldsymbol{V}}{d}_{ij}^{adj}}{\left|\boldsymbol{V}\right|}-\frac{\sum_{j\in \boldsymbol{S}}{d}_{ij}^{adj}}{\left|\boldsymbol{S}\right|} \end{equation*}

The GenePanda score was used to rank all nodes.

#### Improved dual label propagation

Improved dual label propagation (IDLP) was formulated by Zhang *et al.* [4] specially for gene prioritization on gene–disease heterogeneous networks. IDLP involves back-and-forth network propagation on the PGI network and the disease similarity network. Before each propagation, PGI network or disease similarity network is updated with knowledge of GDAs, in the way that genes causing the same diseases get larger edge weight in the PGI network and disease caused by the same genes get larger edge weight in the disease similarity network.

For realization of the IDLP algorithm, the PGI network and disease similarity network were first normalized:

(2.11) \begin{equation*} {\boldsymbol{A}}^{\boldsymbol{norm}}={\boldsymbol{K}}^{-\frac{\mathbf{1}}{\mathbf{2}}}\boldsymbol{A}{\boldsymbol{K}}^{-\frac{\mathbf{1}}{\mathbf{2}}} \end{equation*}

(2.12) \begin{equation*} {\boldsymbol{A}}_{\boldsymbol{DD}}^{\boldsymbol{norm}}={\boldsymbol{K}}_{\boldsymbol{DD}}^{-\frac{\mathbf{1}}{\mathbf{2}}}{\boldsymbol{A}}_{\boldsymbol{DD}}{\boldsymbol{K}}_{\boldsymbol{DD}}^{-\frac{\mathbf{1}}{\mathbf{2}}} \end{equation*}

where ${\boldsymbol{K}}_{\boldsymbol{A}}$ and ${\boldsymbol{K}}_{\boldsymbol{DD}}$ are diagonal matrices with node degrees of PGI network and disease similarity network, respectively.

The IDLP algorithm was realized by repeating the following:

(2.13) \begin{equation*} {\boldsymbol{A}}^{\ast }={\boldsymbol{A}}^{\boldsymbol{norm}}+\gamma \boldsymbol{Y}{\boldsymbol{Y}}^T \end{equation*}

(2.14) \begin{equation*} \boldsymbol{Y}=\beta \left(\boldsymbol{I}-\alpha \right){{\boldsymbol{A}}^{\ast}}^{-1}{\boldsymbol{A}}_{\boldsymbol{PD}} \end{equation*}

(2.15) \begin{equation*} {\boldsymbol{A}}_{\boldsymbol{DD}}^{\ast }={\boldsymbol{A}}_{\boldsymbol{DD}}^{\boldsymbol{norm}}+{\gamma}^{\prime }{\boldsymbol{Y}}^T\boldsymbol{Y} \end{equation*}

(2.16) \begin{equation*} \boldsymbol{Y}={\beta}^{\prime }{\boldsymbol{A}}_{\boldsymbol{PD}}\left(\boldsymbol{I}-{\alpha}^{\prime }{\boldsymbol{A}}_{\boldsymbol{DD}}^{\ast}\right) \end{equation*}

where ${\boldsymbol{A}}^{\ast}$ and ${\boldsymbol{A}}_{\boldsymbol{DD}}^{\ast}$ are the updated PGI and disease similarity networks and $\boldsymbol{Y}$ is the gene–disease relationship matrices to be learnt, which has same dimensions with ${\boldsymbol{A}}_{\boldsymbol{GDA}}$. $\boldsymbol{Y}$ is initialized with random values. The algorithm should be repeated until $\boldsymbol{Y}$ converges. In practice, we performed 20 iterations due to the long runtime of each iteration, caused by the complexity of matrix inverse calculation. Before the iterations, an extra column was added to ${\boldsymbol{A}}_{\boldsymbol{PD}}$ representing cSVD, in which rows for seed genes had value 1; other rows had value 0. The dimension of $\boldsymbol{Y}$ was adjusted accordingly. The final value of the column representing cSVD in $\boldsymbol{Y}$ was used to rank all genes.

## Evaluation of algorithm performance

### Leave-one-out cross-validation

Model performance was internally evaluated using leave-one-out cross-validation (LOOCV). For each repeat of cross-validation, one seed node was left-out from the set of seed genes and the rank of the left-out seed node was used as the performance metric (referred as LOOCV rank). Median and mean values of LOOCV ranks of seed nodes were calculated as overall performance metrics. For methods applied to the gene–disease heterogeneous network, edges between cSVD and the left-out seed gene were also removed to prevent data leaking. Gene ranks given by degree centrality were used as a naive baseline performance.

### Random-seed experiments

Random-seed experiments served to evaluate seed-independent patterns captured by the algorithms. In one trial, a randomly selected 10 seed genes were taken as the input of algorithms and the rank of all nodes were obtained. The experiment was repeated 1000 times, and the median value of the rank of each node across the 1000 experiments was calculated and associated with degree centrality of the nodes. For cSVD-related genes, distributions of the ranks from 1000 experiments were also visualized. The PGI network was used in a random-seed experiment because on the PGI network degree of a node can be directly interpreted in biological sense as the number of interactions a certain gene has.

### External evaluation using MEGASTROKE GWAS results

The MEGASTROKE GWAS [15, 16] of ‘small vessel stroke (SVS) in Europeans’ was used to evaluate the gene prioritization results. SVS is a synonym for cSVD used in the MEGASTROKE study.

Single nucleotide polymorphisms (SNPs) were mapped to genes based on their genomic locations (±1000 bp of the gene region). The genes were indexed with Ensembl gene IDs to remain consistent with the gene prioritization output. *P*-values for all SNPs within the top 200 genes found in the MEGASTROKE summary statistics were extracted. To determine a significance threshold for the genes shortlisted by the algorithms, false discovery rate (FDR)-adjusted *P*-values were calculated for all SNPs. Those genes with a significant proportion of SNPs (determined using a one-sample *t*-test) which passed the FDR-adjusted *P*-value threshold of *P* < 0.05 were considered to be validated within MEGASTROKE.

The MEGASTROKE study identified seven genes associated with the SVS phenotype. We used this list of seven genes as the other way of performance validation. The median rank, number of hits in top 10% of predictions and the list of hits were obtained as performance metrics.

The same validation procedure was applied to degree centrality ranks to obtain a naive baseline performance.

## Results

### Domain knowledge led curation of PGIs

**Figure 2:**
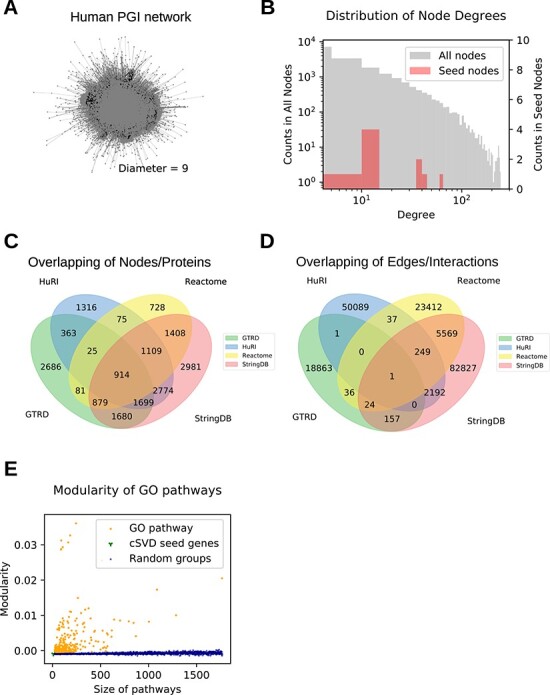
Domain knowledge-guided curation of human protein–gene–disease interaction and assembly of interaction networks. (A) The human PGI network. (B) A heavy tail distribution of degree centrality in PGI network. (C) Venn diagram showing overlaps of genes among databases. (D) Venn diagram showing overlaps of interactions among databases. (E) Modularity of network by GO term ontologies.

The knowledge-lead curation of human PGIs resulted in a PGI network with 18 718 distinct proteins and 183 457 interactions with the largest connected components covering 18 664 proteins (Table 1, Figure 2A). A heavy tail distribution of degrees was observed (Figure 2B). Different PGI databases each have a distinct contribution to the total number of proteins, with a different extent of overlapping between the databases (Figure 2C). The overlap of interactions, however, was to a lesser extent, since biological meaning of interactions from different databases were different (Figure 2D). In particular, only two interactions were found in both HuRI and GTRD databases, which is consistent with the fact that transcriptional regulation mostly does not involve binary interaction between two proteins. To test if the curated network displays known functional properties of PGI networks, modularity scores of GO pathway proteins were calculated and compared to randomly chosen groups of protein. Indeed, higher modularity scores compared to randomly chosen protein groups were observed (Figure 2E).

### Characteristics of cSVD genetic risk factors on human PGI network

**Figure 3:**
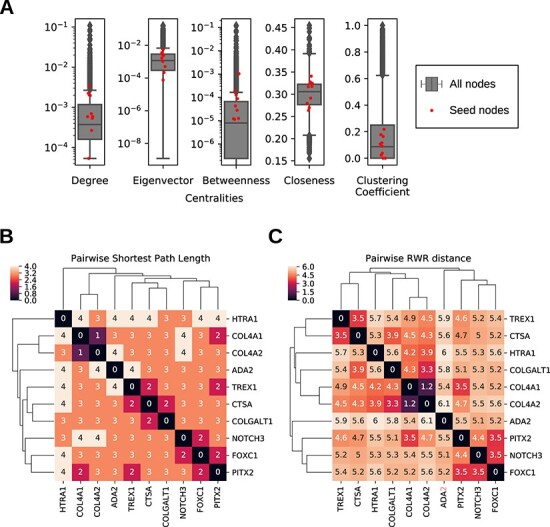
Properties of monogenic risk factors of cSVD in PGI network. (A) Centralities: degree, eigenvector, betweenness, closeness, clustering coefficient. (B) Pairwise distances of seed nodes: shortest path length. (C) Pairwise distances of seed nodes: RWR distance.

To assess the (PGI) network-based properties of known monogenic risk factors (seed genes) of cSVD, we calculated centrality measurements of nodes representing the seed genes. Most (8/10) seed nodes had degree centralities above network median. Six of the seed nodes had eigenvector centralities above the network median. All seed nodes had betweenness centrality above the network median (Figure 3A, Table 3). In addition, six of the seed nodes had a clustering coefficient above the network median.

**Table 3:** Metrics of seed genes in PGI network

**Vertex**	**Degree centrality**	**Eigenvector centrality**	**Betweenness centrality**	**Closeness centrality**	**Clustering coefficient**
TREX1	2.03E-03	1.67E-03	1.91E-04	3.17E-01	3.84E-02
COL4A1	5.88E-04	5.08E-04	2.82E-05	2.79E-01	9.09E-02
COL4A2	5.88E-04	7.54E-05	1.22E-05	2.64E-01	1.64E-01
PITX2	2.24E-03	5.46E-03	1.27E-04	3.41E-01	1.16E-01
FOXC1	6.95E-04	3.59E-03	1.26E-05	3.25E-01	2.18E-01
NOTCH3	1.98E-03	2.43E-03	8.82E-05	3.17E-01	1.07E-01
HTRA1	2.67E-04	2.18E-04	1.14E-05	2.70E-01	0.00E+00
ADA2	5.34E-05	1.03E-03	0.00E+00	2.91E-01	0.00E+00
CTSA	3.42E-03	2.57E-03	1.04E-03	3.27E-01	2.28E-02
COLGALT1	5.34E-04	3.07E-03	4.29E-05	3.25E-01	1.78E-01
Seed median	6.41E-04	2.05E-03	3.56E-05	3.17E-01	9.88E-02
Seed mean	1.24E-03	2.06E-03	1.55E-04	3.06E-01	9.34E-02
Graph median	3.74E-04	1.17E-03	7.99E-06	3.06E-01	8.67E-02
Graph mean	1.05E-03	2.84E-03	1.27E-04	2.99E-01	1.83E-01

To know the relative positions of seed nodes in the human PGI network, pairwise distances of seed nodes defined by shortest path length or RWR were calculated (Figure 3B–C). COL4A1 and COL4A2 genes were two of the six subunits of the type IV collagen and were, therefore, neighbours. PITX2, NOTCH3 and FOXC1 genes, all of which are involved in NOTCH signaling pathway, formed another cluster. The lysosome biogenesis regulator gene, TREX1, clustered with CTSA gene, which is a lysosome peptidase. COLGALT1 was clustered with either CTSA or loosely with the COL4A1/COL4A2 cluster, depending on the distance metric used. ADA2 and HTRA1 were not in proximity with any other seed nodes in the human PGI network.

### Evaluation of network-based gene prioritization methods on cSVD with LOOCV

Performance of gene prioritization algorithms was firstly evaluated with LOOCV. On the PGI network, RWR had the best performance with a median LOOCV rank of 1356.5 in seed nodes, followed by N2V with a median rank of 2165 (Table 4). DIAMoND and GenePanda failed to achieve comparable performance. Next, we evaluated algorithms that were applicable on the protein/disease heterogeneous network. RWRH achieved the best performance with a median LOOCV rank of 185.5 in seed nodes, followed by N2VH with a median rank of 820.5. Performance of the IDLP algorithm was not comparable to RWRH and N2VH. Performance of RWR and N2V algorithms was both dramatically improved by using the heterogeneous network.

**Table 4:** Performance of algorithms in LOOCV

	**LOOCV rank**							**Rank**
	**RWR**	**N2V**	**DIAMOnD**	**GenePanda**	**RWRH**	**N2VH**	**IDLP**	**Degree centrality**
TREX1	3006	3907	na^a^	5408	1429	4539	18 103	2643.5
COL4A1	29	20	2344	22	21	19	18 201	7505.5
COL4A2	30	20	177	5	31	21	16 574	7505.5
PITX2	523	2035	na	4698	78	131	236	2326
FOXC1	1515	2157	na	3652	54	126	15 323	6792.5
NOTCH3	1198	4710	na	10 439	76	816	18 925	2727
HTRA1	8731	1871	269	14 636	293	825	949	11 076.5
ADA2	15 307.5	10 750	na	9380.5	18 206	15 555	10 011	17 226
CTSA	1037	3951	na	4418	993	3427	17 603	1255
COLGALT1	3302	2173	1960	4393	6197	4632	10 016	7923
Median	1356.5	2165	na	4558	185.5	820.5	15 948.5	7149
Mean	3467.85	3159.4	na	5705.15	2737.8	3009.1	12 594.1	6698.0

^a^The DIAMOnD algorithm is a stepwise prioritization algorithm. The maximum steps were set to 5000, so that LOOCV rank of some seed genes is not available (na).

Patterns could be observed on the variance of LOOCV ranks of seed genes. Seed genes, which belonged to clusters defined by network-based distance measures, tended to have better LOOCV rank. COL4A1 and COL4A2 were ranked in the top 40 genes in most algorithms. NOTCH3, FOXC1 and PITX2 had better ranks in two RWR-based algorithms. Seed genes with higher degree centrality, like CTSA, NOTCH3 and PITX2, also tended to have better LOOCV rank in the RWR algorithm.

### Random-seed experiments

**Figure 4:**
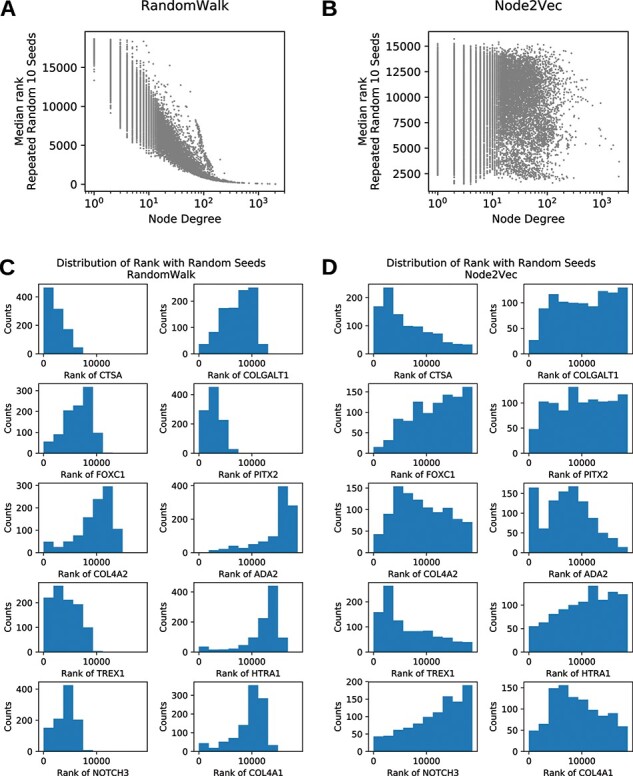
Dependency of rank on node degree. (A) Degree dependency of median random-seed node rank (all nodes) in RWR. (B) Degree dependency of median random-seed node rank (all nodes) in N2V. (C) Distribution of ranks of seed genes in random-seed experiments on RWR. (D) Distribution of ranks of seed genes in random-seed experiments on N2V.

Contributions to results of gene prioritization come from seed-dependent (choice of seeds) and seed-independent sources (intrinsic properties of the network). Here, we sought to study the influence of degree centrality on gene prioritization results. The implications were explained in detail in the Discussion section. To measure the dependency on degree centrality, we simulated 1000 experiments with 10 randomly selected seed genes. Association of the median rank of each gene in 1000 simulations with degree centrality of the node representing the gene was examined. For RWR, lower median rank of a gene in random-seed experiment was associated with higher degree centralities, meaning that nodes with higher degree centrality got better rank regardless of chosen seed genes (Figure 4A). The same trend was not observed for N2V (Figure 4B). We then took a deeper look at the distribution of rank for seed genes of cSVD in random-seed simulations. In RWR, ranks of seed genes from the random 10-seed experiment were narrowly distributed, where in N2V, the distribution was wider (Figure 4C and D).

### External validation of gene prioritization results in MEGASTROKE GWAS

We next validated the top 200 predictions of the algorithms with MEGASTROKE GWAS results. The top predictions of GenePanda had the most genes that could be validated in GWAS (90/200), followed by N2VH and N2V (70/200 and 53/200; Table 5). RWRH achieved the best rankings for the seven confirmed SVS-associated genes from the MEGASTROKE study (median rank 1840 with 4/7 among top 10% predictions) (Table 5). Like the observation in LOOCV, more GWAS-validated genes in the top 200 predictions were observed for RWR and N2V algorithms when the heterogeneous network was used. Including disease–gene association in the network improved the performance in ranking GWAS-confirmed genes for RWR but not for N2V. The full list of prioritized genes and their significance in MEGASTROKE GWAS can be found in the Supplementary Table S2 and S3.

**Table 5:** Validation of gene prioritization results by MEGASTROKE GWAS

	**SNP**	**Gene**	**Sig. genes in GWAS**		
**Algorithm**	**(Sig./total)**	**(Sig./total)**	**Median rank**	**Hits@10%**	**Hits list**
RWR	411/3110	25/200	5251	2/7	ICA1L, SEMA4A
N2V	1139/3027	53/200	4526	1/7	SEMA4A
DIAMOnD	47/2649	3/200	na	0/7	
GenePanda	1859/2918	90/200	8544	1/7	ZCCHC14
RWRH	784/2767	45/200	1840	4/7	SH3PXD2A, SEMA4A, ICA1L, ZCCHC14
N2VH	1597/2590	70/200	5979	1/7	SH3PXD2A
IDLP	603/3700	41/200	9318.5	1/7	ZCCHC14
Degree centrality	385/2144	31/200	7697	0/7	

## Discussion

In the current study, we applied network-based gene prioritization algorithms to shortlist new candidate genes for cSVD. A domain knowledge-lead curation of PGIs was done as the input network. To select the most suitable algorithm, we benchmarked seven algorithms and observed good performance for RWRH and N2VH in LOOCV. Given the total number of genes and proteins in the heterogeneous network (19 463), the median ranks of rediscovery in LOOCV for RWRH (185.5) translate to 50% of disease-causing genes enriched in the top 0.95% (185.5/19 463) of candidate genes. In the following tests of the two algorithms, we found that N2V algorithm was less prone to pick up seed-independent patterns. External validation of the algorithms using MEGASTROKE GWAS identified several genes within the top 200 candidate genes that were associated with small vessel stroke, indicating that there is a certain degree of agreement between network-based algorithms and GWAS. The PGI network and the pipeline for algorithm benchmarking were made available online (https://github.com/huayu-zhang/gp-bench).

Network-based prioritization algorithms are based on different assumptions and mechanisms. If the assumptions or the mechanisms do not fit with the underlying biology and genetic basis of diseases, we will observe suboptimal performance of the algorithms. The overall assumption of the algorithms that are benchmarked in this article is the ‘guilt-by-association’ principle [23]. In a biomedical sense, the principle can be approximately translated to ‘a gene which interacts with known disease-causing genes has a better chance to be a potential disease-causing gene’. This assumption is partly true, if we consider that genes work as components of biological pathways and functional modules [1]. Therefore, variations in one of the components could lead to similar disease phenotypes. However, the assumption does not cover situations in which none of the genes belonging to a responsible pathway or functional module is known or if the genetic structure of the disease is more sporadic than clustered. Indeed, clustered (according to network-based distance metrics) genes tend to have better LOOCV rank in our study. The IDLP algorithm additionally assumes the smoothness of the adjacency matrix (edge weights smoothed by network propagation) of the PGI network and disease similarity network, which is not necessarily true. During the derivation of the algorithms, certain diseases were normally taken as example cases, demonstrating the (superior) performance of the algorithm. It is possible that the mechanism of certain algorithms fits better with the genetic structure of the example disease. For example, the DIAMOnD algorithm finds next candidate genes among the neighbours of the seed gene set updated to the current step, which naturally favours diseases with disease-causing genes forming large clusters on the PGI network in rediscovery analysis. Indeed, the DIAMOnD algorithm had superior performance over RWR in lysosomal storage disease, of which the disease-causing genes have one of the highest *z*-score for forming connected components [7]. As for cSVD, the seed genes are in different clusters or isolated, explaining poor performance for the DIAMOnD algorithm in LOOCV. In summary, we would recommend comparison of multiple algorithms before network-based gene prioritization methods are applied to a certain disease. In addition, combination of multiple methods (or ‘ensemble’ from the machine learning term) may help to cancel out intrinsic bias of a single algorithm. Relevant research has been done for breast cancer [24], but the subsequent issue on how ensemble methods should be chosen would require a systemic study.

The PGI network can be supplemented with disease–gene interactions and disease–disease similarity relationships to create a heterogeneous disease–gene network. It was previously found that using the heterogeneous network improved performance of some network-based gene prioritization methods [24, 25]. However, such findings have not been confirmed for network embedding algorithms such as N2V. In our experiment, we observed substantial improvement of performance in LOOCV for N2V using the heterogeneous network, indicating that N2VH could also utilize information of existing disease–gene associations to infer new ones.

The choice of data sources for the input network plays an important role in network-based gene prioritization methods. Previous studies relied on either single curated PGI databases or curation of multiple sources [7, 26]. We believe that the source of PGI should be carefully selected for several reasons: (i) reliability of estimation of algorithm performance in LOOCV can be influenced by the degree centrality (number of interactions of a node) of seed nodes. We demonstrated that, for algorithms like RWR and its variations, LOOCV ranks of nodes were positively correlated with their degree centralities, regardless of choice of seed nodes. In other words, performance of RWR-like algorithms would appear to be better in LOOCV just by having higher degree centralities for the seed nodes, which does not necessarily reflect true ability for the algorithm to predict new candidate genes. Since known disease-associated genes tend to attract more research interests, including literature-based PGIs would disproportionately increase degree centrality of seed genes, resulting in over-estimation of RWR-like algorithms. In this article, we attempted to avoid such over-estimation by preferably curating PGIs obtained from high-throughput methods, where the chance of a gene to be researched was not based on researchers’ own interests. However, we could not completely exclude other experimental evidence curated in the STRING database, due to the need to include all seed genes in the PGI network. (ii) Careful choice of data sources enables discoveries of poorly researched genes. Like the argument in the previous point, the systematically higher ranks of high degree genes mean prioritization would be biased towards well-researched genes, if data sources subjected to researchers’ interest are included. On the other hand, omics data from high-throughput methods are not subjected to the bias towards well-researched genes, giving poorly researched genes a fair chance to be prioritized. (iii) Different types of interactions have different biological meanings. Controlling the source of PGI makes it possible to utilize different types of interactions in gene prioritization, although few currently available network-based algorithms allow this.

Given the possible over-estimation of performance assessment by LOOCV, we also validated the algorithm using GWAS results. Since all approaches are limited in their own ways in identification of disease-associated genes, it is not possible to evaluate the performance of algorithms against the hypothetical ‘ground truth’. Nonetheless, we could see either if top predictions of the algorithms overlap with genes with significantly correlated SNPs from GWAS or if better ranks are observed for GWAS-confirmed genes. All algorithms but DIAMOnD and RWR had more validated genes in top 200 predictions than the naive predictions by degree centrality. RWRH, N2V, N2VH and RWR algorithms had better median rank of seven GWAS-confirmed genes than the expected median. RWRH and N2VH algorithms both contained the *SH3PXD2A* gene in their top 200 predictions. This gene has been found to be associated with any stroke and SVS in MEGASTROKE (at genome-wide significance and suggestive significance, respectively) and with periventricular hyperintensity in brain MRI imaging [25, 26]. In addition, the RWRH algorithm included four of the seven GWAS-confirmed genes in top 10% of prediction. These observations suggest that these models are able to capture the biological mechanisms involved in SVS and shortlist candidate genes that could be used to develop a greater understanding of the pathophysiology of SVS, despite the room for improvement in both reliability and precision.

There are several limitations of this study. Firstly, this study only compared selected network-based gene prioritization methods for cSVD, so that, for example, machine learning-based algorithms were not included. Secondly, the methods applied in this study did not utilize or (in the off-the-shelf form) did not allow the use of other information, such as other types of omics data like tissue-specific gene expression. Thirdly, network-based gene prioritization tools take the concept of a gene or a protein as the base entity, while, in reality, a gene or a protein involves a cascade of complex biological activities, such as splicing, transcriptional regulation, translational regulation, etc. The current methods need improvement to both incorporate the complexity of the information and to increase the resolution of entities (e.g. to a base pair in the genome). Thirdly, GBA-based algorithms rely on the prior information given by seed genes, which means these algorithms will not perform well for diseases with no or limited known associating genes or when the known disease-associating genes do not form a homogenous cluster. In such case, using genes in relevant functional pathways as seed genes provides another chance to use network-based algorithm for gene prioritization. To integrate the extension of including genes in a relevant functional pathway as seed information, future studies need to determine how pathway genes can be integrated (as seed genes or as new relationships in the graph) and the implications of different strategy. Finally, a reliable validation method for benchmarking the algorithms is still lacking, as we reasoned that the LOOCV was prone to over-estimation of the performance and results of GWAS will not cover all disease-associated genes by nature. Future work should aim at tackling these limitations to improve the performance and reliability of network-based gene prioritization algorithms.

**Key Points**

- Random walk with restart with disease–gene heterogeneous network has overall better performance for application in cerebral small vessel disease despite its susceptibility to bias caused by degree centrality.
- Choice of network-based gene prioritization methods should be made for the target disease since the performance of these methods is disease dependent.
- We provide the integrated pipeline to benchmark commonly used algorithms for quick start of algorithm comparison and evaluation.
- Network gene prioritization methods based on ‘guilt-by-association’ principle are unlikely to find disease-associated genes outside the functional clusters of currently known ones.

## Data availability statement

The curated gene protein interaction data and disease gene association data underlyting this article are available in the gp-bench github repository at https://github.com/huayu-zhang/gp-bench. The datasets were derived from sources in the public domain: HuRI (http://www.interactome-atlas.org/), GTRD (https://gtrd.biouml.org/), Reactome (https://reactome.org/), String (https://string-db.org/), Mimminer (https://www3.cmbi.umcn.nl/MimMiner/help.html) and DisGeNET (https://www.disgenet.org/). The GWAS validation data from MEGASTROKE project are available at https://www.megastroke.org/. 

## Funding

Medical Research Council and Health Data Research UK (MR/S004149/1 to H.W., K.S., K.R. and H.Z.); Engineering and Physical Sciences Research Council (K.S.); Economic and Social Research Council (K.S.); National Institute for Health Research (England) (K.S.); Chief Scientist Office of the Scottish Government Health and Social Care Directorates (K.S.); Health and Social Care Research and Development Division (Welsh Government) (K.S.); Public Health Agency (Northern Ireland) (K.S.); British Heart Foundation (K.S.); Wellcome (K.S.); Industrial Strategy Challenge (MC_PC_18029 to H.W. and H.Z.); Wellcome Institutional Translation Partnership Award (PIII054 to H.W. and H.Z.); Medical Research Foundation (G.R.).

## Supplementary Material

supplementary_tables_bib-20-0744_bbab006

## Notes

**Huayu Zhang** is a research fellow working in the Centre for Medical Informatics, University of Edinburgh, with a background in both biomedical sciences and bioinformatics. He is interested in biomedical knowledge representation and integration and the use of integrated data sources for predictions.

**Amy Ferguson** is a research fellow in statistical genetics.

**Grant Robertson** is a PhD candidate in the Armstrong Group, School of Informatics University of Edinburgh.

**Muchen Jiang** is a 5th-year medical student at the University of Edinburgh.

**Teng Zhang** is a research and development officer in the University of Hongkong.

**Cathie Sudlow** OBE FRSE is a neurologist and professor of Neurology and Clinical Epidemiology. She is the director of British Heart Foundation Data Science Centre.

**Keith Smith** is a UKRI innovations fellow in health data science. His work focuses on theory and clinical applications of complex neural and biological networks. He has a PhD in biomedical signal processing from the University of Edinburgh, a Masters in Actuarial Science from Heriot-Watt University, and a Bachelors in Mathematics from the University of Glasgow.

**Kristiina Rannikmae** is a Health Data Research UK Rutherford Senior Clinical Research Fellow in the University of Edinburgh. Her research is developing methods for identifying clinical phenotypes from routinely collected health data with applications to stroke genetics. She also works part time as a neurology consultant in NHS Forth Valley.

**Honghan Wu** is a lecturer at Institute of Health Informatics, University College London, with a PhD in computing science and leads the KnowLab group: https://knowlab.github.io/. He is a Rutherford research fellow of Health Data Research UK. His current research interest is in using text technologies and knowledge graph techniques for health data research.
